# Open partial nephrectomy for entirely intraparenchymal tumors: a matched case-control study of oncologic outcome and complication rate

**DOI:** 10.1590/S1677-5538.IBJU.2016.0040

**Published:** 2017

**Authors:** Piotr Zapala, Bartosz Dybowski, Nina Miazek, Piotr Radziszewski

**Affiliations:** 1 Department of Urology, Medical University of Warsaw, Poland

**Keywords:** Kidney, Neoplasms, Nephrectomy

## Abstract

**Purpose:**

To compare the oncologic and clinical outcomes for open partial nephrectomy (OPN) performed in patients with entirely intraparenchymal tumors versus case-matched controls, with exophytic lesions.

**Material and methods:**

Patients having undergone OPN between 2007 and 2012 were investigated. Exclusion criteria included patients with a benign tumor, advanced malignancy, malignancies other than renal cell carcinoma, end-stage renal failure, or 3 or more co-existing chronic diseases. Individuals with tumors that were invisible at the renal surface were identified, and then matched with 2 controls chosen for tumor size, pathology, age, follow-up period, and presence of a solitary kidney. Oncological status, perioperative, and postoperative data were collected and compared between groups.

**Results:**

17 individuals with entirely endophytic RCC tumors and available oncologic status were identified. For five patients, only one suitable control could be identified, bringing the control group number to 29. All tumors were clear cell carcinomas staged at pT1a. Median tumor size was 25mm for endophytic lesions, and 27mm for exophytic masses (P=0.32). The operative period was extended by 20 minutes for intrarenal tumors (P=0.03), with one case of a positive surgical margin in each group (P=0.7). There were no significant differences in perioperative or postoperative complications. Median follow-up was 47 and 43 months for patients with endophytic and exophytic tumors respectively. Disease recurrence was recorded in one patient after endophytic tumor resection, and in four controls (P=0.4).

**Conclusions:**

OPN shows equivalent safety and efficacy for both intrarenal RCC tumors and exophytic tumors of the same size and type.

## INTRODUCTION

In 2012, renal cell carcinoma (RCC) represented the nineth most common malignancy worldwide (1). Radical nephrectomy (RN) was considered the gold standard for RCC treatment but stage migration, advances in surgical technique, and an increased appreciation of the morbidity associated with renal insufficiency, have resulted in an expansion of the indications for nephron-sparing techniques. Partial nephrectomy (PN) for localized T1 RCC has an oncologic outcome similar to that of radical surgery, and incurs in a minimal risk of postoperative renal insufficiency (2-5). According to the guidelines of both the European Association of Urology and American Urological Association, PN is strongly recommended for patients with T1a tumors.

Despite these clear recommendations, PN utilization is variable and depends on a number of factors including the physician’s preference, surgical skills, and tumor characteristics. It has been emphasized by many authors that PN of hilar and endophytic tumors is associated with a higher complication risk. The feasibility of performing a partial nephrectomy, in the case of an entirely endophytic tumor that does not extend to the renal surface, is of particular concern. These cases of tumor localization pose several challenges to surgeon, including the intraoperative identification of the tumor and its extent. The aim of this study was to compare the oncologic and clinical outcomes of PN for completely intraparenchymal tumors, with case-matched controls operated for exophytic lesions. To the best of our knowledge, this study is the first to compare results of nephron-sparing techniques for tumors not extending to the renal surface, with a matched control group of exophytic masses.

## MATERIALS AND METHODS

### Patient Selection and Outcome Measurements

Patients with a renal mass who underwent PN from 2007 to 2012, in a single department, were reviewed. Those with benign tumors, advanced malignancies other than RCC, a contralateral tumor, end-stage renal failure, or three or more co-existing chronic diseases, were excluded from the study. Within the patient group termed “ENDO”, with entirely endophytic RCC tumors, tumors were defined as renal masses localized exclusively in the parenchyma of the kidney, and macroscopically invisible at the kidney’s surface during surgery. Each of these patients was then matched with two others, with tumors visible at the renal surface (termed group EXO). The investigated group (ENDO) was matched with controls for age (±15 years), pathological subtype, clinical stage, tumor size (+/-1.5cm), time of surgery (same year), and type of indication for PN (elective vs. absolute).

Patient data were gathered for specified demographic and clinical variables, tumor characteristics, perioperative, and oncologic outcomes. Pathologic data comprised histological subtype, stage (assigned according to the 2009 TNM classification system) (6), grade (7), and surgical margins (8). Perioperative outcomes included clamping time, length of stay (LOS), transfusion, and surgical complications graded according to the Clavien - Dindo classification (9). A trifecta defined as warm ischemia time <25 minutes, negative surgical margins, and no perioperative complications was also calculated. Postoperative follow-up included ultrasound or CT imaging, performed every six months. Oncologic status for this study was assessed using CT performed at least 2 years after surgery. If any suspicion of disease progression arose, a complete restaging for further treatment was performed.

### Surgical Technique

PN was performed using a lateral retroperitoneal approach in all patients. In the endophytic group, the renal parenchyma was incised over the tumor after identification of the mass by intraoperative US or palpation. The renal pedicle was always visualized and prepared for clamping. If necessary, warm ischemia was applied by clamping the main artery or its specific branch. Wedge resection, sometimes combined with enucleation, was utilized. Vessels were unclamped as soon as the major source of bleeding was found and controlled. Otherwise a standard procedure was applied (10-14).

### Statistical analyses

Continuous variables were presented as medians accompanied by ranges or interquartile ranges (IQR). Differences between groups were evaluated using the U Mann-Whitney test for continuous variables, and by the chi-square test for categorical variables. As follow-up was inconsistent, with a six-month time span for control imaging, the precise time until recurrence could not always be reliably ascertained. For this reason, recurrence-free survival (RFS) was estimated using the modified Kaplan-Meier method for interval-censored data. Difference in survival between the endophytic group and the controls was assessed using the log-rank test. For all statistical analyses, a 2-sided P value <0.05 was considered statistically significant. Statistical analyses were performed using STATISTICA 12 (StatSoft, USA).

## RESULTS

During the reported period, 313 patients underwent PN in the department, with seventy-eight individuals excluded according to the criteria detailed earlier. Among the remaining 235 patients, we identified 24 individuals with entirely endophytic RCC tumors. For 7 of these patients, their oncological status was unavailable at the time of the study, leading to their exclusion. The final ENDO group therefore comprised 17 patients, with a median follow-up of 47 months (IQR 34). For five of these patients, we could identify only one control that matched all of our criteria. As a result, the control group comprised 29 patients, with a median follow-up of 43 months (IQR 23) (P=0.51).

All tumors in both groups were clear cell RCC, stage pT1a. Five patients, two with endophytic and three with exophytic tumors, had solitary kidneys. Other absolute indications for PN were not identified in the studied groups. Median tumor size and distribution showed no significant differences (Table-1). Six endophytic lesions (35.3%) and 4 exophytic tumors (13.8%) were 11-20mm, 3 endophytic tumors (17.6%) and 14 exophytic tumors (48.3%) were 21-30mm, while 6 (35.3%) endophytic tumors and 11 (37.9%) exophytic tumors were 31-40mm. Tumor localization was comparable for both groups: nine (52.9%) endophytic tumors and 13 (44.8%) exophytic tumors were entirely polar (P=0.6). A hilar location was found in 2 (11.8%) endophytic tumors, and 7 (31.8%) exophytic tumors (P=0.31). Also, there were no significant differences between the groups in terms of age, indication, and health status (Table-1).

**Table 1:** Demographic and clinical characteristics of the patients who underwent open partial nephrectomy for entirely endophytic or exophytic renal tumors.

Variable	ENDO	%	EXO	%	P
n	17		29		
Age	61 (11)		63 (9)		0.95
Sex (Male/Female)	10/7		19/10		0.65
ASA score:					0.4
1 and 2	13	76.5	25	86.2	
3	4	23.5	4	13.8	
Hypertension	10	58.8	9	31	0.06
Diabetes	2	11.8	4	13.8	0.8
Preoperative serum creatinine (mg/dl)	1 (0.3)		1 (0.3)		0.99
Tumor size (mm)	25 (14)		27 (11)		0.3
Laterality (right)	9	52.9	11	37.9	0.3
Solitary kidney	2	11.8	3	10.3	0.9

**ENDO =** endophytic tumors; **EXO =** exophytic tumors; **ASA =** American Society of Anesthesiologists Qualitative data are presented as median (interquartile range)

Surgery in the ENDO group took approximately 20 minutes longer (P=0.03) and was performed more frequently under ischemia, than in the EXO group (P< 0.01; Table-2). If the artery was clamped, ischemia time never exceeded 20 minutes, with median values for both groups of 12 min. For localization of the endophytic tumors, in 7/17 cases intraoperative ultrasound was used, with the aid of a radiologist. Oncologic status was determined between January 2014 and April 2015. In both groups, all patients were alive at the time of the study. There were also no significant differences in perioperative and postoperative complications (P=0.8 and P=0.6 respectively). The renal collecting system was opened and repaired in 1 patient with an endophytic tumor and in 3 patients from the control group. Only one (6%) postoperative complication was observed in the ENDO group (wound infection and ileus, Clavien 2), with 3 events (10%) in the EXO group (hypokalemia and hypertension, Clavien 1; transient ischemic attack, Clavien 2; reoperation due to bleeding from intercostal artery, Clavien 3). No perioperative, cardiovascular life-threatening incidents occurred. Blood transfusions were carried out for 2 patients in the ENDO group, and 3 patients in the control group; each transfusion required 2 units of packed red blood cells. Positive surgical margins were found for 1 (6%) endophytic tumor, and 1 (3.4%) exophytic tumor (P=0.7). Disease recurrence was detected in one patient from the ENDO group (local relapse), and in four patients from the EXO group: two patients suffered a local relapse, one patient developed a multifocal recurrence in the ipsilateral kidney, and one presented with metastasis to the adrenal gland. The difference in recurrence-free survival was insignificant (P=0.3). In two patients from the EXO group (both with a solitary kidney), and in one patient from the ENDO group, creatinine levels exceeded normal values throughout the follow-up period. However, the median values for serum creatinine were unchanged for both groups during follow-up (Tables 1 and 2).

**Table 2:** Perioperative and postoperative outcomes for patients having undergone open partial nephrectomy for entirely endophytic or exophytic renal tumors.

Variable	ENDO	%	EXO	%	P
n	17		29		
Operative time (minutes)	120 (34)		101 (35)		0.03
Renal artery clamped	9	52.9	4	13.8	<0.01
Warm ischemia time (minutes)	12 (3)		12.5 (7.5)		0.9
Intraoperative US use	7	41	0	0	<0.0002
PSM	1	6	1	3.4	0.7
LOS (days)	9 (34)		9 (23)		0.4
Trifecta	15	88.2	25	86.2	0.8
Follow-up (months)	47 (34)		43 (23)		0.5
Disease recurrence	1	5.9	4	13.8	0.4
Serum creatinine in follow-up (mg/dL)	0.9 (0.2)		1 (0.4)		0.96

**ENDO =** endophytic tumors; **EXO =** exophytic tumors; **US =** ultrasound; **PSM** = positive surgical margin; **LOS =** length of stay

Qualitative data are presented as median (interquartile range)

## DISCUSSION

Despite explicit guideline recommendations, radical nephrectomy remains the most widely used treatment for T1 RCC tumors (15). Moreover, high tumor complexity diminishes the use of PN, even at high volume academic centers, from frequencies of 75-100% for cases with low nephrometry scores, to 0-45% for those with high scores (16). Frequent use of radical nephrectomy seems to be the result of concern about possible complications and poor surgical benefits. When considering the usefulness of nephrometry as a predictive tool, we believe that it is essential not to overestimate an endophytic location when predicting PN outcome. Despite the additional challenge presented by these tumors, entirely intrarenal T1a tumors should always be considered as candidates for the nephron-sparing approach. The extent of adhesion to the renal collecting system is of more importance than tumor visibility alone. Therefore, when considering operative options, and candidacy for PN, we consider not only the absolute indications and staging, but principally the relationship of the tumor to the hilar structures. Hilar T1 tumors that dislocate vessels and/or the renal collecting system are therefore usually disqualified from PN.

Demographic and pathologic data did not differ significantly between cases and controls, which confirmed that our exclusion criteria and the matching process were adequate. There were no statistically significant differences in terms of positive surgical margins or recurrence. The length of stay was also similar for cases and controls. Although renal vessels were clamped significantly more often during endophytic PN, the warm ischemia times were comparable, and within safe limits.

The longer operative time for PN could be attributed both to vessel dissection and to the use of ultrasound. In cases where small exophytic tumors were localized far from the hilum, we could perform PN with neither artery clamping, nor artery dissection. This meant that the whole procedure, from skin incision to closure, could take as little as 60 minutes. In almost half of the cases of intraparenchymal lesions, intraoperative ultrasound was used to localize the tumor site, while in other cases, parenchymal bulging, especially when examined in ischemia, was the guiding parameter. Calling on the assistance of the radiologist prolongs surgery, with additional time needed to examine the kidney intraoperatively, along with logistic issues. Additionally, requesting the assistance of the radiologist when the surgeon does not have adequate ultrasound experience can cause delays, which can be avoided should the urologist be familiar with the apparatus. Although not proven statistically, our experience was that the smaller the intraparenchymal tumor, the more challenging it was to find. Additionally, appropriate patient selection and technical skills could adequately prevent major complications such as fistula formation. Transfusion was required infrequently in both groups. Only minor complications occurred, and no significant difference in total complication event number was noted.

We are, however, aware of some limitations of the study. Our study cohort was small, which impacts statistical power. Moreover, even with data-censoring, inconstant follow-up remains a significant limitation. Additionally, its retrospective nature renders our analyses prone to selection bias. Contrary to some reports (17, 18), the intrarenal tumors in our material were localized predominantly to the poles. This could be explained by selection bias or by the fact that in the majority of previous studies, endophytic lesions were defined as all those extending intrarenally by >50% of their diameter. Indeed the lack of any significant differences in relation to polar line and entirely endophytic versus exophytic masses has already been described (19).

Initial studies of the use of an open nephron-sparing treatment for endophytic lesions suggested that the resection of intrarenal RCC tumors was associated with longer ischemic times and higher peak creatinine levels in the immediate postoperative period (20, 21). On the other hand, initial laparoscopic procedures led to the concern that endophytic tumors were associated with a higher complication rate (22), and that corticomedullar growth patterns were the most significant predictor of postoperative complications (23). Contemporaneous with the development of minimally invasive PN, several reports have now confirmed similar outcomes for open (24, 25) versus robotic PN of complex tumors (18, 25-27). Authors have emphasized the low rate of major complications (19, 25, 26), oncological safety (19, 25, 26), short time of warm ischemia (19, 26), and superior preservation of renal function (24); all of which are in line with our data. The introduction of particular management measures and tools, such as intraoperative US (28), which is now strongly advised for complex tumors, may also result in improved outcomes. In terms of contemporary series on endophytic renal tumors and robot assisted partial nephrectomy (19), the surgical management of such lesions is technically extremely challenging. Therefore, open surgery remains the best option for countries and regions where surgical robots are unavailable. Under such circumstances, the low morbidity and favorable oncologic data presented in our study, would lead us to advocate the use of partial nephrectomy over radical nephrectomy.

## CONCLUSIONS

Our results confirm that an open approach for PN of endophytic T1a tumors is both safe and efficient, and should also be considered for patients with lesions located entirely interstitially. This localization, for small renal masses, carried no increased risk of complication or recurrence, and should not be incorporated in nephrometry scores.

## LIST OF ABBREVIATIONS

RCC: Renal cell carcinoma
RN: Radical nephrectomy
PN: Partial nephrectomy
LOS: Length of stay
CT: Computed tomography
IQR: Interquartile ranges
RFS: Recurrence-free survival
US: Ultrasound
